# Protective effects of Equisetum arvense methanolic extract on sperm characteristics and in vitro fertilization potential in experimental diabetic mice: An experimental study

**DOI:** 10.18502/ijrm.v18i2.6415

**Published:** 2020-02-27

**Authors:** Mehrsa Fajri, Abbas Ahmadi, Rajabali Sadrkhanlou

**Affiliations:** Department of Basic Sciences, Faculty of Veterinary Medicine, Urmia University, Urmia, Iran.

**Keywords:** Diabetes, Equisetum, Sperm, Streptozotocin, Mice.

## Abstract

**Background:**

Diabetes mellitus is a metabolic disorder characterized by impaired insulin secretion or the inability of tissues to respond to insulin. This disease can damage the testis and reduce semen quality. Therefore, it can impair the potential for male fertility. Different herbal therapeutic treatments have been used to control diabetes and its complications.

**Objective:**

This study aimed to evaluate the effects of streptozotocin-induced diabetes on sperm and in vitro fertilization (IVF) potential and investigate the protective effects of Equisetum arvense methanolic extract on diabetic mice.

**Materials and Methods:**

Twenty-four adult male mice were divided into four groups: control-sham, diabetic, diabetic + Equisetum extract (250 mg/kg), and diabetic + Equisetum extract (500 mg/kg). After 45 days, sperm samples were collected from the cauda epididymis to evaluate the characteristics of sperm (including viability, count, motility, morphology and chromatin/DNA integrity of sperm) and the IVF potential.

**Results:**

Sperm motility and viability were increased remarkably (p ≤ 0.001) in the treated groups compared with the non-treated diabetic group. The decrease in sperm count in the diabetic group compared with the treated groups was not significant. Moreover, the percentage of sperm with DNA damage, nuclear immaturity, and abnormal morphology was decreased significantly (p ≤ 0.001) in the treated groups compared with the diabetic group. The treated animals exhibited remarkably higher fertilization rates and a higher percentage of fertilized oocytes that developed toward the blastocyst stage compared with the non-treated diabetic group (p ≤ 0.001).

**Conclusion:**

The methanolic extract of the Equisetum arvense inhibited diabetes-induced detrimental effects on sperm quality and fertilization rate, which may have been associated with hypoglycemic and antioxidative activities in this plant.

## 1. Introduction

Male infertility accounts for 30%-40% of overall infertility and is usually related to abnormal sperm production or function. A number of diseases, such as diabetes mellitus, can induce impairment in male fertility potential (1). Diabetes mellitus is a metabolic disorder of carbohydrates, fats, and proteins (2) that is characterized by impaired insulin secretion or a decrease in tissue sensitivity to insulin, leading to hyperglycemia (3). Diabetes causes arteriosclerosis, microangiopathy, nephropathy, and neuropathy. It is thought that neuropathy and vascular insufficiency may lead to impotence, decreased libido, and ejaculation disorder (4). It has also been shown that diabetes alters spermatogenesis, steroidogenesis, and sperm maturation (5). The consequences of this alteration are reduction in sperm quantity, motility, and viability (1); damage to sperm DNA that leads to decreased embryo quality and implantation rates (6); structural defects of sperm; and reduced ability to penetrate oocytes (5). Overall, these effects can lead to a reduction in male fertility through its effects on the male reproductive system (5).

As many of the chemical drugs used to control diabetes have side effects, the use of plants has become an appealing alternative, as plant-based products generally have low production costs and fewer side effects. There are many plants with hypoglycemic activity that can be used for such purposes (7), including Equisetum arvense L. (Equisetaceae, subgenus Equisetum, traditional name: horsetail). Equisetum arvense is a well-known plant that is distributed throughout the northern hemisphere and contains many different compounds, such as minerals, flavonoids, phenolic glycosides, alkaloids, and triterpenoids (8). It has been shown that the methanolic extract of Equisetum arvense has hepatoprotective, antioxidant (9), and antidiabetic effects (10). So, this extract is useful to control diabetes and its complications (11).

Separate studies have reported on the adverse effects of diabetes on male fertility and on the antidiabetic effects of Equisetum arvense methanolic extract. However, no previous studies have evaluated the effects of Equisetum arvense on the characteristics of sperm and the fertility potential in diabetes. Thus, the purpose of this study was to evaluate the protective effects of Equisetum arvense on the quality and quantity of sperm and on the fertility potential in diabetic mice.

## 2. Materials and Methods

This study was conducted at the Faculty of Veterinary Medicine, Urmia University.

### Preparation of Equisetum arvense methanolic extract 

Dried aerial parts of the plant were powdered using an electric grinder, and the powder (500 gr) was packed in a filter paper and placed into a Soxhlet apparatus and extracted with methanol (Merck, Germany). The methanol was then evaporated in a rotary evaporator, and the obtained extract was stored at -20 °C until further use (10).

### Animals and induction of experimental diabetes

In this study, 24 adult male mice (6-8 wk old, 20-25 gr) were provided by the laboratory animal center of the Faculty of Veterinary Medicine, Urmia University. The animals were maintained in a controlled environment (12-h dark/light cycle, temperature of 22 °C ± 2 °C, and humidity of 30%-60%) and were provided food and water ad libitum.

In order to induce diabetes, 50 mg/kg body weight streptozotocin dissolved in sodium citrate buffer (0.1 M, pH 4.5) was injected intraperitoneally to fasting mice for 5 days. Blood glucose concentration was measured after 5-7 days and mice with blood glucose levels ≥250 mg/dL were considered diabetic (2). These mice were classified into four groups:

• Control-sham group (received intraperitoneal injection of sodium citrate buffer (0.1 mL) for 5 days)
• Diabetic group (after the induction of diabetes, mice were kept for 45 days)
• Diabetic group treated with methanolic extract at a dose of 250 mg/kg body weight
• Diabetic group treated with methanolic extract at a dose of 500 mg/kg body weight

In the third and fourth groups, after the induction of diabetes, the mice received extract orally daily for 45 days. The mice of the first and second groups received normal saline orally daily for 45 days.

The doses of methanolic extract were selected according to previous studies (10, 11).

### Sperm preparation

After 45 days, the mice were anesthetized and euthanized by intraperitoneal injection of ketamine and xylazine. The cauda epididymides were then removed and transferred into a 30-mm Petri dish containing 1 mL of pre-equilibrated human tubal fluid medium (HTF medium; Sigma-Aldrich, St. Louis, USA) with 4 mg/mL of bovine serum albumin (BSA; Sigma-Aldrich, St. Louis, USA). After the epididymides were minced by making 5-7 slashes using a 30-gage needle of an insulin syringe and incubated in an atmosphere of 5% CO${}_{2}$ at 37 °C for 30 min, spermatozoa swum out of the epididymal tubules. After washing, the motile sperm were incubated at 37 °C under 5% CO${}_{2}$ for 1 h for capacitation (12).

### Oocyte collection and in vitro fertilization (IVF)

To prepare the ovum, three non-diabetic adult female mice (6-8 wk old) for each male mice were superovulated with an injection of 7.5 UI pregnant mare serum gonadotropin (PMSG; Folligon, Netherlands) intraperitoneally and injection of 7.5 UI human chorionic gonadotropin (hCG; Folligon, Netherlands) 48 hr later. The mice were then anesthetized and sacrificed by cervical dislocation 12 h after the injection of hGC. The oviducts were removed and placed in pre-equilibrated HTF medium (incubated in 5% CO${}_{2}$ at 37 °C) containing 4 mg/mL BSA. The oocytes were then removed from the oviducts by dissection. After washing, they were transferred into fertilization drops under mineral oil in HTF medium with 4 mg/mL BSA. The capacitated motile sperm were then added at 10${}^{6}$/mL culture medium and incubated in 5% CO${}_{2}$ at 37 °C for 5-6 h (13).

After incubation, the fertilized oocytes (zygotes) with two pronuclei were transferred into fresh HTF-BSA medium. The number of two-cell embryos was counted 24 h after culture of zygotes. Also, the number of arrested embryos and blastocysts was counted after 120 h under an inverted microscope. The arrested embryos were classified into the three following groups (14):

Type I: lysed, fragmented, and necrotic embryos completely

Type II: embryos with partially fragmented and lysed blastomeres

Type III: embryos with some fragmented and lysed blastomeres and/or cytoplasmic vesicles

### Sperm count

To determine the sperm count, 190 μL of distilled water was poured into a 1-mL microcentrifuge tube, to which 10 μL of epididymal sperm was then added. So, a 1:20 dilution was prepared. Then, 10 μL of the diluted specimen was transferred into the hemocytometer, and the number of sperms was counted under a light microscope with a magnification of ×400 (12).

### Sperm motility

To evaluate sperm motility, one drop of the sperm suspension was poured into a glass slide and covered with a coverslip. The number of motile sperm was then counted under a light microscope with a magnification of ×200 (12).

### Sperm viability

To determine sperm viability, 20 μL of sperm suspension was mixed with 20 μL of 1% eosin solution, and after 20-30 s, 20 μL of 5% nigrosin solution was added. After the preparation of smears and drying slides, the percentage of dead (pink) and live (discolored) sperm was calculated under a light microscope with a magnification of ×400 (12).

### Sperm morphology

In order to investigate sperm morphology, aniline blue staining was used for counting abnormal sperm by appearance. Also, eosin-nigrosin staining was used for counting sperm containing cytoplasmic residue (12).

### Sperm chromatin maturation test

In order to assess sperm nucleus maturation, after the preparation of smears from sperm suspensions and drying of slides, samples were fixed in 3% glutaraldehyde in saline phosphate buffer for 30 min and stained with aniline blue solution (AB) for 5 min. Finally, the slides were evaluated under a light microscope with a magnification of ×100. The sperm with immature nuclear chromatin were stained dark blue, and those with mature nuclear chromatin were stained pale blue (15).

### Sperm DNA damage

Acridine orange (AO) staining was used to evaluate the sperm DNA damage and detect double- and single-stranded regions of sperm chromatin. Briefly, air-dried smears were fixed in Carnoy's fixative for at least 2 h and stained with AO solution for 10 min. After rinsing with deionized water and drying, the slides were observed using a fluorescence microscope with a 460-nm filter. Sperm with normal double-stranded DNA appeared green, whereas sperm with single-stranded DNA produced yellow to red fluorescence (15).

### Ethical consideration

All experimental protocols were approved by the ethical committee of Urmia University based on proven principles for laboratory animal care (code: IR-UU-AEC-1475/AD3/).

### Statistical analysis

SPSS software (version 16, SPSS Inc., Chicago, USA) and one-way ANOVA were used to analyze the data. To determine significant differences between groups, Tukey's *post hoc* test with a threshold of p < 0.05 was used. Also, IVF data was analyzed by using Minitab software and 2 Proportion with p < 0.05. All data were expressed as mean ± SD.

## 3. Results

### Sperm motility

Evaluation of sperm motility revealed that the percentage of motile sperm was reduced significantly (p ≤ 0.001) in the diabetic group compared with the control-sham group, whereas this parameter was increased significantly (p ≤ 0.001 and p ≤ 0.001) in the treated groups compared with the diabetic group. There was no significant difference (p = 0.234) between the two treated groups (Table I).

### Sperm viability

**Figure 1:**
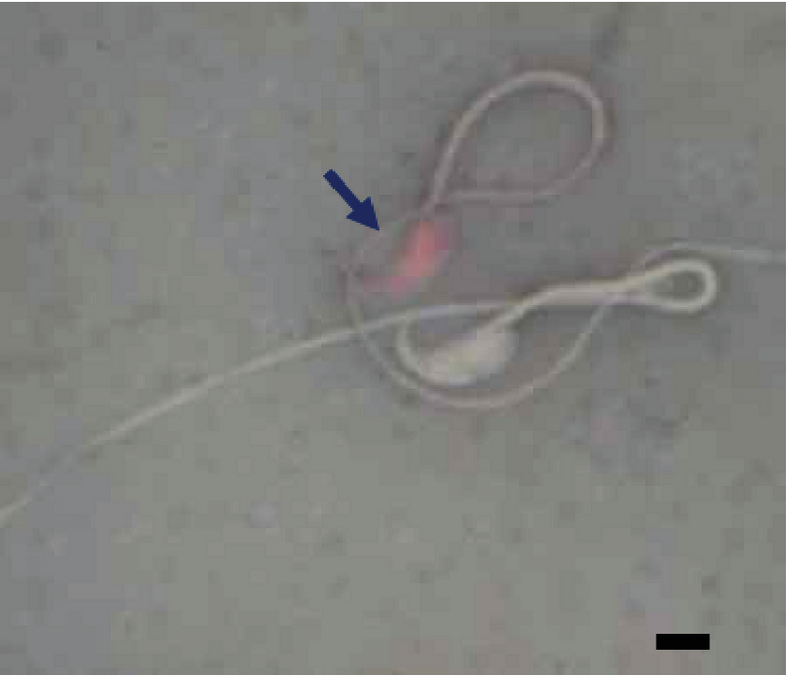
Microscopic view of sperms. The blue arrow indicates the dead sperm into which the eosin stain has penetrated into the cytoplasm (Eosin-Y-nigrosin staining, ×1,000). Scale bar = 2.8 µm.

Sperm viability was decreased significantly (p ≤ 0.001) in the diabetic group compared with the control-sham group. On the other hand, sperm viability was significantly higher (p ≤ 0.001) in the treated groups than in the diabetic group (Figure 1, Table I).

### Sperm count

Our results reveal a decrease in the number of cauda epididymis sperms in the diabetic group compared with the other groups, but this decrease was not statistically significant (Table I).

### Sperm morphology

The percentage of sperm with abnormal morphology in the diabetic group was increased significantly (p ≤ 0.001) compared with the control-sham group, whereas this parameter was significantly (p ≤ 0.001) lower in treated groups than in the diabetic group. No significant difference was observed (p = 0.581) between the treated groups (Table I).

### Sperm chromatin maturation test

**Figure 2:**
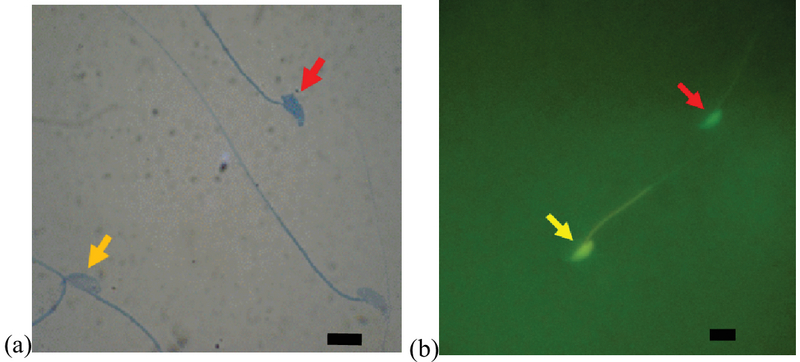
(a) Light microscope view of sperms with mature chromatin (pale blue head: yellow arrow) and immature chromatin (dark blue head: red arrow) (AB staining, ×1000). Scale bar = 5.6 µm. (b) Fluorescence microscope view. Red arrow: sperm head with normal DNA is green; Yellow arrow: sperm head with damaged DNA is yellow (AO staining, ×1,000). Scale bar = 5.6 µm.

The evaluation of sperm chromatin maturation revealed that the percentage of sperm with immature chromatin was significantly higher (p ≤ 0.001) in the diabetic group than in the control-sham group, whereas the percentage of sperm with immature chromatin showed a significant decrease (p ≤ 0.001) in the treated groups compared with the diabetic group. Also, there was no significant difference (p = 0.087) between the treated groups (Figure 2, Table I).

### Sperm DNA damage

We observed a significant increase in the percentage of sperm DNA damage in the diabetic group compared with the control-sham group (p ≤ 0.001), whereas this parameter was reduced significantly (p ≤ 0.001) in the treated groups compared with the diabetic group (Figure 2, Table I).

### Fertilization rates and embryonic development

**Figure 3:**
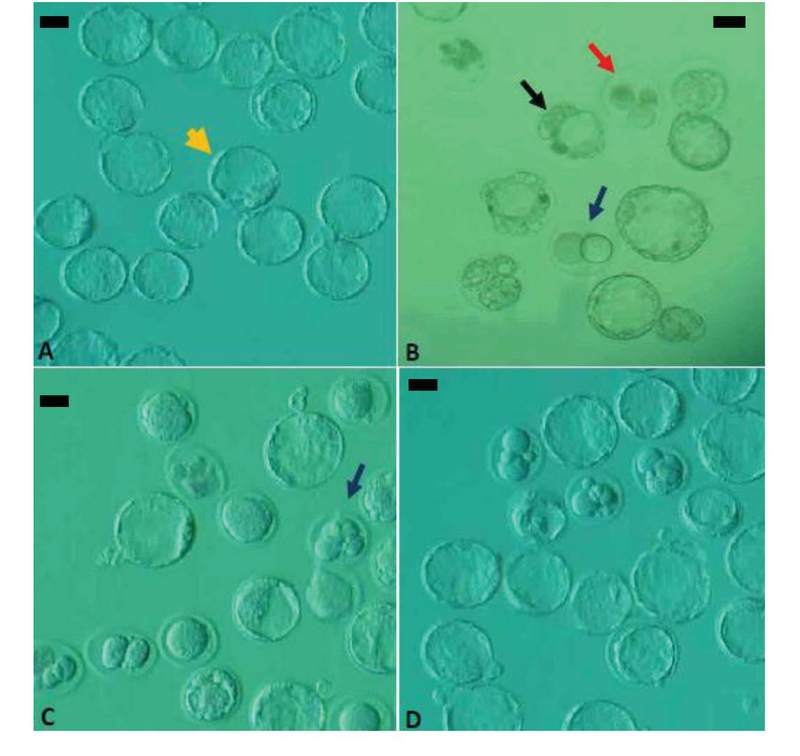
The results of IVF and embryonic development. (A) Control-sham group, the yellow arrow indicates the advanced blastocyst. (B) Diabetic group, the black arrow indicates the early blastocyst with abnormal morphology, the blue arrow indicates the arrested two-cell embryo type I, and the red arrow indicates the arrested four-cell embryo type II. (C) D + EE250 group, the blue arrow indicates the arrested four-cell embryo type I. (D) D + EE500 group (inverted microscope, ×100). Scale bar = 35 µm.

The results of IVF and embryonic development in the different groups are presented in Figure 3 and Table II. Our findings reveal that the fertilization rate was decreased significantly (p = 0.003) in the diabetic group compared with the control-sham group, whereas this parameter was significantly (p = 0.006) higher in the treated group (with a dose of 500 mg/kg) than in the diabetic group. The percentage of two-cell embryos showed a significant decrease (p ≤ 0.001) in the diabetic group compared with the control-sham group. On the other hand, the percentage of two-cell embryos was increased significantly (p = 0.025) in the treated group (with a dose of 500 mg/kg) compared with the diabetic group. A significant decrease (p ≤ 0.001) in the overall percentage of blastocysts was observed in the diabetic group compared with the control-sham group, but the overall percentage of blastocysts was significantly (p ≤ 0.001) higher, respectively, in the D + EE500 and D + EE250 groups than in the diabetic group. In the diabetic group, the percentage of arrested embryos was increased significantly (p ≤ 0.001) compared with the control-sham group. This parameter in the treated groups was significantly (p ≤ 0.001) lower in the diabetic group.

Our observations also revealed that most arrested embryos in the diabetic group were type I arrested embryos, whereas in the treated groups, most arrested embryos were type III arrested embryos (p = 0.001 and p ≤ 0.001).

**Table 1:** Sperm parameters in different groups


**Factors**	**Groups**			
	**Control-sham**	**Diabetic**	**Diabetic + EE250**	**Diabetic + EE500**
**Sperm motility (%)**	62.6 ± 3.36	32.8 ± 4.20${}^{a}$	54.4 ± 4.82${}^{b}$	48.2 ± 6.72${}^{ab}$
**Sperm viability (%)**	88 ± 3.39	36.2 ± 4.14${}^{a}$	58.6 ± 4.09${}^{ab}$	68 ± 3.60${}^{abc}$
**Sperm count (×10${}^{6}$)**	63.2 ± 3.34	52.4 ± 11.45	59.6 ± 19.66	79.6 ± 47.7
**Sperm morphology (% abnormalities)**	5.8 ± 1.92	69.4 ± 6.42${}^{a}$	27.2 ± 5.21${}^{ab}$	23.4 ± 3.78${}^{ab}$
**Nuclear immaturity (%)**	2.8 ± 1.48	25.8 ± 5.11${}^{a}$	7 ± 1.58${}^{b}$	2.4 ± 1.14${}^{b}$
**Sperm DNA damage (%)**	1.8 ± 1.48	44.6 ± 8.20${}^{a}$	14.2 ± 4.20${}^{ab}$	11.6 ± 4.21${}^{ab}$
All data are expressed as Mean ± SD ${}^{a}$Significantly different from the control-sham group (p < 0.05), ${}^{b}$Significantly different from the diabetic group (p < 0.05), and ${}^{c}$Significantly different from the diabetic + EE250 group (p < 0.05)				

**Table 2:** The results of IVF and embryonic development in different groups


**Factors**	**Groups**			
	**Control-sham**	**Diabetic**	**Diabetic + EE250**	**Diabetic + EE500**
**Total number of oocytes**	176	249	290	265
**Fertilization rate**	156 (88.63)	194 (77.91)${}^{a}$	205 (70.68)${}^{a}$	231 (87.16)${}^{c}$
**Two-cell embryo rate**	153 (98.07)	159 (81.95)${}^{a}$	181 (88.29)${}^{a}$	207 (89.61)${}^{ab}$
**Blastocyst rate**	90 (57.69)	48 (24.74)${}^{a}$	98 (47.80)${}^{b}$	114 (49.35)${}^{b}$
**Arrested embryo rate**	66 (42.30)	146 (75.25)${}^{a}$	107 (52.19)${}^{b}$	117 (50.64)${}^{b}$
**Type I arrested embryos**	4 (2.56)	111 (57.21)${}^{a}$	18 (8.78)${}^{ab}$	27 (11.68)${}^{ab}$
**Type II arrested embryos**	11 (7.05)	25 (12.88)	31 (15.12)${}^{a}$	45 (19.48)${}^{a}$
**Type III arrested embryos**	51 (32.69)	10 (5.15)${}^{a}$	58 (28.29)${}^{b}$	45 (19.48)${}^{abc}$
Data are expressed as n (%), a: Significantly different from the control-sham group (p < 0.05), b: Significantly different from the diabetic group (p < 0.05) and c: Significantly different from the diabetic + EE250 group (p < 0.05)				

## 4. Discussion

The results of this study indicate that diabetes had detrimental effects on sperm parameters (including viability, count, motility, and morphology), chromatin/DNA integrity of sperm, IVF outcome, and embryonic development, and oral administration of Equisetum arvense methanolic extract reduced these diabetes-induced detrimental effects.

AO and AB staining were used to evaluate sperm DNA integrity and sperm nucleus maturation. Despite limitations, such as observer subjectivity, heterogeneous slide staining, long duration of fixation (in the case of AO staining), and microscopic nature of these assessments, the methods have advantages, including simplicity and low cost, and these methods are still used as efficient tools for evaluating sperm chromatin structure in a wide variety of basic and clinical studies (16). In the present study, regarding AB and AO staining, the percentage of sperm with an immature nucleus and damaged DNA was increased significantly in the diabetic group compared with the control-sham group. In agreement with our findings, Bahmanzadeh *et al*. (17) revealed that streptozotocin-induced diabetes in rats had detrimental effects on the characteristics of sperm and nuclear chromatin integrity. Previous studies have revealed that hyperglycemia leads to increased production of reactive oxygen species (ROS) and causes oxidative stress in different tissues (18). According to these findings, oxidative stress affects the integrity of DNA in the sperm nucleus. In the process of spermatogenesis, DNA-attached histones are replaced by protamines. The packaging of sperm DNA by protamine protects it from free radical attack (19, 20). Based on our findings and those of previous studies, after the induction of diabetes, sperm chromatin was not fully compacted, and the DNA in these sperm was more sensitive to oxidative stress. Also, after the induction of diabetes, free radicals can directly damage sperm DNA by attacking its purine and pyrimidine bases (19).

In the current study, the evaluation of sperm morphology revealed that the percentage of abnormal sperm was increased significantly in the diabetic group compared with the control-sham group, in accordance with prior results (17, 21). Previous studies revealed that diabetes can impair the process of spermatogenesis in men (22). It has been reported that when spermatogenesis is damaged, cytoplasmic extrusion mechanisms cannot act normally. Therefore, the sperm with cytoplasmic droplets (excess residual cytoplasm) are released from the germinal epithelium. These sperm are immature and are defective in function (23).

Previous studies have revealed that oxidative stress considerably deteriorates the sperm membrane by lipid peroxidation. Accordingly, controlled peroxidation of lipids and the integrity of sperm membrane play an important role in sperm viability (18, 20). Our results reveal that the percentage of dead sperms was increased significantly in the diabetic group compared with the control-sham group, which was consistent with previous findings (17, 21).

In the present study, sperm motility was reduced significantly in the diabetic group compared with the control-sham group. In accordance with our results, Arikawe *et al*. (24) revealed that all of the sperm parameters were significantly reduced in rats with Alloxan-induced diabetes compared with control rats. Reduction in sperm motility may be related to ROS overproduction and diffusion of the free radicals across the membranes into the sperm. The sperm membrane is vulnerable to ROS stress because it contains large amounts of unsaturated fatty acids, such as docosahexaenoic acid, which has six double bonds per molecule (25).

In the present study, the number of sperm was decreased in the diabetic group compared with the control-sham group. An increase in the production of free radical impairs the function of Sertoli and Leydig cells, leading to a decrease in synthesis and secretion of testosterone. Decreasing these hormone levels leads to interference in spermatogenesis and reduction in sperm count (26). The results of a present study on sperm count were in agreement with that of the aforementioned study (21).

Previous studies revealed that the fertilization rate and fraction of fertilized embryos developing to the blastocyst stage were markedly decreased in diabetic mice compared with control mice (5). Our results in IVF and embryonic development were in agreement with those of the aforementioned study. The number of fertilized zygotes, two-cell embryos, and blastocysts was decreased significantly in the diabetic group compared with the control-sham group. Also, there was a significant increase in the number of arrested embryos in the diabetic group compared with the control-sham group. It has been reported that embryonic development both *in vivo* and in vitro is associated with sperm quality (27). The overproduction of ROS in seminal fluid causes infertility by changing the structure of sperm DNA, increasing the percentage of dead sperm, and disconnecting sperm from the surface of oocytes (28). Also, an increase in ROS levels disrupts the fluidity of the sperm membrane, leading to a decrease in sperm motility and impairment of membrane fusion events, such as sperm-oocyte fusion (29).

In the present study, we revealed that the methanolic extract of Equisetum arvense is able to inhibit some complications of diabetes. It has been reported that the methanolic extract of this plant has antidiabetic effects, and it is useful for controlling diabetes (10, 11). According to previous studies, the methanolic extract of Equisetum arvense has antioxidative activity, and it can be used as a source of natural antioxidants (30).

Our observations revealed that the percentage of sperm with an immature nucleus and damaged DNA in the treated groups was decreased significantly compared with the diabetic group. In the treated groups, the extract of Equisetum arvense probably produced a hypoglycemic effect and reduced the production of free radicals. As a result, sperm DNA was packaged by protamines and protected from free radical attack.

The percentage of abnormal and dead sperm was significantly lower in the treated groups than in the diabetic group. This decrease may be related to hypoglycemic and antioxidative activities in the extract of Equisetum arvense, which consequently leads to decreased lipid peroxidation.

In the present study, a significant increase in sperm motility and count was observed in the treated groups compared with the diabetic group. The extract of Equisetum arvense, probably through a reduction in ROS production, reduced diabetes-induced damage and increased the motility of sperm. In addition, the reduction in free radicals led to enhanced Leydig and Sertoli cell physiological activities and increased the sperm count.

The results of IVF and embryonic development revealed that the rate of fertilization, two-cell embryos, and blastocysts was increased significantly in the treated groups compared with the diabetic group. Also, the fraction of arrested embryos was decreased significantly in the treated groups compared with the diabetic group. These results may be related to antioxidant activity in the extract of Equisetum arvense.

To our knowledge, there is no previously published data about the efficacy of Equisetum arvense methanolic extract in the protection of male reproductive system disorders in diabetic mice. Thus, we are not able to compare these findings with those of other studies.

## 5. Conclusion

It can be concluded that diabetes reduces fertilization rates by decreasing sperm quality. However, the methanolic extract of Equisetum arvense, as a hypoglycemic and antioxidative compound, reduces diabetes-induced damage to sperm, thereby leading to an increase in the rate of IVF and a decrease in the fraction of arrested embryos.

##  Conﬂict of Interest

The authors have no conﬂicts of interest to declare with regard to this work.
